# Supplementary material for: Short and long sleeping mutants reveal links between sleep and macroautophagy
Source: eLife. 2021 Jun 4;10:e64140. doi: 10.7554/eLife.64140 (PMC8177895; doi:10.7554/eLife.64140)
Supplement: Transparent reporting form [file elife-64140-transrepform1.docx]

***eLife’s* transparent reporting form**

We encourage authors to provide detailed information *within their submission* to facilitate the interpretation and replication of experiments. Authors can upload supporting documentation to indicate the use of appropriate reporting guidelines for health-related research (see [EQUATOR Network](http://www.equator-network.org/%20)), life science research (see the [BioSharing Information Resource](https://biosharing.org/)), or the [ARRIVE guidelines](http://www.plosbiology.org/article/info:doi/10.1371/journal.pbio.1000412) for reporting work involving animal research. Where applicable, authors should refer to any relevant reporting standards documents in this form.

If you have any questions, please consult our Journal Policies and/or contact us: [editorial@elifesciences.org](mailto:editorial@elifesciences.org).

**Sample-size estimation**

- You should state whether an appropriate sample size was computed when the study was being designed
- You should state the statistical method of sample size computation and any required assumptions
- If no explicit power analysis was used, you should describe how you decided what sample (replicate) size (number) to use

Please outline where this information can be found within the submission (e.g., sections or figure legends), or explain why this information doesn’t apply to your submission:

Replicate numbers were determined from experience with the techniques performed. Ns reflecting this are reported throughout the figure legends.

Behavior: we generally aimed for N’s in the 20-30s, except where poor yields limited our N’s (mostly re: aus combinations with transgenes). In this case, we verified results with multiple different approaches (see Response to Reviewers).

Imaging: since we trained our machine learning algorithm on brains selected as representative of the complete dataset, we estimated the likely N needed to resolve potential effects by eye during imaging, plus factoring in results from previous live-imaging experiments where that information was available.

**Replicates**

- You should report how often each experiment was performed
- You should include a definition of biological versus technical replication
- The data obtained should be provided and sufficient information should be provided to indicate the number of independent biological and/or technical replicates
- If you encountered any outliers, you should describe how these were handled
- Criteria for exclusion/inclusion of data should be clearly stated
- High-throughput sequence data should be uploaded before submission, with a private link for reviewers provided (these are available from both GEO and ArrayExpress)

Please outline where this information can be found within the submission (e.g., sections or figure legends), or explain why this information doesn’t apply to your submission:

Ns are reported in each figure legend, individual datapoints are plotted on most graphs, and individual values are included in Source Data files linked to each figure. We did not exclude statistical outliers; any data excluded was for obvious confounds that corrupted the data. (ex: brains badly damaged during dissection prior to imaging or that shifted far off-center during imaging; flies that died midway through a sleep assay or, in one case, where we erroneously recorded sleep from flies in the wrong age range)

**Statistical reporting**

- Statistical analysis methods should be described and justified
- Raw data should be presented in figures whenever informative to do so (typically when N per group is less than 10)
- For each experiment, you should identify the statistical tests used, exact values of N, definitions of center, methods of multiple test correction, and dispersion and precision measures (e.g., mean, median, SD, SEM, confidence intervals; and, for the major substantive results, a measure of effect size (e.g., Pearson's r, Cohen's d)
- Report exact p-values wherever possible alongside the summary statistics and 95% confidence intervals. These should be reported for all key questions and not only when the p-value is less than 0.05.

Please outline where this information can be found within the submission (e.g., sections or figure legends), or explain why this information doesn’t apply to your submission:

(For large datasets, or papers with a very large number of statistical tests, you may upload a single table file with tests, Ns, etc., with reference to sections in the manuscript.)

We modified our figures during revision to directly report all relevant comparisons’ exact p-values, in place of the traditional asterisk/NS notation. Medians and interquartiles are shown on graphs where they were informative and practical. Statistical tests and Ns are reported in figure legends. Individual datapoints are shown in figures where it was practical to do so, and we show representative micrographs for all of our live-imaging experiments.

**Group allocation**

- Indicate how samples were allocated into experimental groups (in the case of clinical studies, please specify allocation to treatment method); if randomization was used, please also state if restricted randomization was applied
- Indicate if masking was used during group allocation, data collection and/or data analysis

Please outline where this information can be found within the submission (e.g., sections or figure legends), or explain why this information doesn’t apply to your submission:

NA for most experiments, where we are looking solely at genotype differences. In cases where animals of the same genotype were assigned to multiple drug groups, timepoints, or other non-genetic manipulations, allocation was random.

**Additional data files (“source data”)**

- We encourage you to upload relevant additional data files, such as numerical data that are represented as a graph in a figure, or as a summary table
- Where provided, these should be in the most useful format, and they can be uploaded as “Source data” files linked to a main figure or table
- Include model definition files including the full list of parameters used
- Include code used for data analysis (e.g., R, MatLab)
- Avoid stating that data files are “available upon request”

Please indicate the figures or tables for which source data files have been provided:

Source data files are directly linked to our figures for all numerical data. Parameters and analysis methods are indicated in figure legends or methods.
